# Characterization of an artificial skull cap for cranio-maxillofacial surgery training

**DOI:** 10.1007/s10856-018-6143-4

**Published:** 2018-08-17

**Authors:** Marianne Hollensteiner, David Fürst, Peter Augat, Falk Schrödl, Benjamin Esterer, Stefan Gabauer, Stefan Hunger, Michael Malek, Daniel Stephan, Andreas Schrempf

**Affiliations:** 1 Research Group for Surgical Simulators Linz, Upper Austria University of Applied Sciences, Linz, Austria; 2 Institue of Biomechanics, Berufsgenossenschaftliche Unfallklinik Murnau and Paracelsus Medical University Salzburg, Murnau, Germany; 3 Institute of Anatomy, Paracelsus Medical University, Salzburg, Austria; 4 Department for Oral and Maxillofacial Surgery, Kepler University Hospital, Med Campus III, Linz, Austria; 5 W & H Austria GmbH, Bürmoos, Austria

## Abstract

Cranial grafts are favored to reconstruct skeletal defects because of their reduced resorption and their histocompatibility. Training possibilities for novice surgeons include the “learning by doing” on the patient, specimens or simulators. Although the acceptance of simulators is growing, the major drawback is the lack of validated bone models. The aim of this study was to create and validate a realistic skull cap model and to show superiority compared to a commercially available skull model. Characteristic forces during machinery procedures were recorded and thickness parameters from the bony layers were obtained. The thickness values of the bone layers of the developed parietal bone were comparable to the human ones. Differences between drilling and sawing forces of human and artificial bones were not detected using statistical analysis. In contrast the parameters of the commercially available skull model were significantly different. However, as a result, a model-based simulator for tabula externa graft lift training, consisting of a brain, skull bone cap and covering soft tissues was created. This simulator enables the training of all procedural steps of a “split thickness graft lift”. In conclusion, an artificial skull cap suitable for parietal graft lift training was manufactured and validated against human parietal bones.

## Highlights

- Axial tool insertion forces were identified as suitable parameter to validate haptics of artificial bone materials compared to human bone.
- Poylurethane edited with mineral fillers and blowing agents can realisitcally mimic skull bones.
- *μ*CT images prove the realistic thickness of all bone layers.
- Realistic haptic performance of artificial skull caps confirmed their suitability for training in the field of cranio-maxillofacial surgery.

## Introduction

Calvarial bone grafts are used for the reconstruction of skeletal defects after trauma, tumor, infection or congenital pseudarthrosis. Their demand increased since the first reported cranioplasty in 1668 [1] and these autologous grafts are harvested about 2.2 million times per year worldwide [2]. Cranial grafts are more favored in contrast to grafts from other donor sites due to several reasons: a hidden scar under the hairline, pain reduction of the donor site in comparison to other sites, a rapid vascularization time [3] and thus less resorption of the graft (only 17 to 20%) [4, 5]. Further, large amounts of cortical bone are harvestable [6] and autologous skull grafts are resistant against infections, are histocompatible, non-immunogenic and there are hardly any risks of transferring diseases compared to allogeneous grafts [1, 7]. A common method to harvest parietal grafts is the split thickness graft method. With this procedure, the harvest of outer cortex strips of 2 × 6 cm is possible. After a skin incision and the retraction of the overlying soft tissues, the outline of the graft is drawn with a drill. Further, a trough is formed to flatten the edges of the “bone island”. An oscillating saw is used to cut the bone into smaller strips and to cut the remaining diploic connections below the graft. To intersect the remaining diploic connections, a sharp flexible osteotome, and eventually, slight taps of a mallet, are used [8]. The risks of this surgical procedure include bleeding, subdural hematomas, paresthesia, intracranial injury, dural tear and even the death of the patient [3, 4]. In order to avoid these complications and to minimize the risks for the patient an intensive training is necessary [9] which allows not only practicing for the procedure itself but also for a suitable handling of the surgical devices and the application of appropriate surgical machinery speeds and forces [10].

Since already more than 100 years, the surgical education and training still follow the Halstedian approach: Surgical residents first watch a procedure, assist and take part in internships before they are allowed to make first hands on experiences under supervision of an experienced surgeon [11]. Other training possibilities include human and animal specimens, live animals and simulators [12]. While the use of specimen, human as well as animal, or live animals is banned in some countries due to ethical concerns [10] drawbacks of simulators are costs, fidelity and validity issues [13]. Nevertheless, well designed and validated physical models provide a realistic haptic feedback [14] which enhances learning and the transfer of surgical skills [15–19]. Further, model-based simulators reduce the need for expensive human or animal specimens. Additionally, the training possibilities for novice surgeons are nearly unlimited [20] and simulators are able to document the learning progress [21]. In sight of the aforementioned points, it is clear that the acceptance of simulators as a training opportunity is rapidly growing [9]. Commercially available biomechanical models are usually made of polyurethane (PU) following the standard ASTM F1839 [22] and are very often considered as the gold standard for orthopedic testing and training. These commercially available artificial bones often reflect biomechanical properties of human bone [23, 24]. However, they are not suitable for surgical training since they do not create bone-like haptics during orthopedic simulations [25–27]. Thus, new bone materials mimicking realistic haptics during simulated orthopedic interventions are necessary and should provide a realistic haptic feedback during the model-based simulation. The aim of this study was to design a novel synthetic skull cap and to determine, if the skull cap would provide realistic machining haptics and bone structure compared to human parietal bone for tabula externa graft lift training. The artificial skull bone imitating the anatomical bone layers of inner and outer cortices as well as the diploe was made of a PU material mixture, which was already identified as suitable in a former study [28].

## Materials and methods

### Customized artificial skull cap

**Fig. 1:**
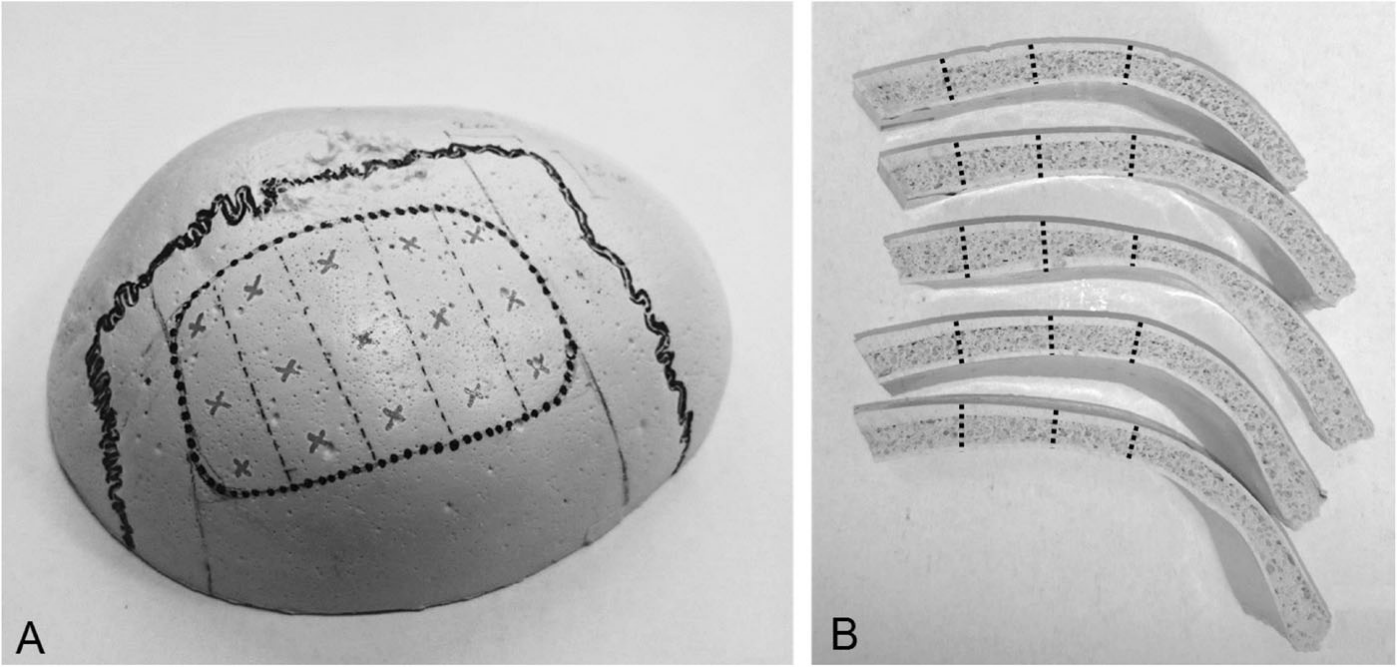
Customized skull cap **a** and cut parietal slices of the left hemisphere **b**. Measurement locations were marked with an “x” a and perpendicular drilling pathways were marked with a dotted line **b**

To manufacture an artificial skull cap (ASC), a two-part rectangular silicone-based mold with the shape of a human skull cap was used. The created skull caps had a height of approximately 7 cm and an elliptical base dimension of 16 × 13 cm. The materials used to create the ASC were described in detail in a former study [28] mainly consisting of PU resin, mineral fillers, cell stabilizers and water as blowing agent. A fixed amount of the liquid material mixture for the outer table was poured in the part of the silicone mold which represents the outer surface of the human skull cap. The liquid material was automatically swiveled within a rotational molding machine (EVM Rotationsgußmaschine, Kaupo Plankenhorn e.K., Spaichingen, Germany) to enable a uniform distribution. After 3 min, the mold was closed and the mixture for the cancellous diploic layer was injected with a syringe. Due to the addition of water, which reacted with the isocyanate of the PU resin, carbon dioxide emerged, forming a PU foam. The foam expanded during the curing process and resulted in a complete fill-out of the closed silicone mold, thus creating the diploic bone of the skull cap. Excessive foam and formed gases were able to leave the closed mold by outgasing holes. After 3 min curing, the mold was opened and a smaller amount of the cortical material was poured onto the newly formed artificial diploic bone. Again, the liquid was swiveled for 3 min. Following an additional curing time of one hour, the skull cap could be removed from the mold. Since the materials were not completely cured during their assembly, the different layers created remained interconnected. Thus, the skull cap imitates the three structures of a human calvarial bone, i.e. tabula externa, diploe and tabula interna. The sagittal, coronoidal and lambdoidal sutures were identified and marked with a black pen (full thick line, see Fig. 1a) on the cured skull cap model. According to Kohan et al. [29] and Abubaker et al. [30] a safe area for parietal graft lift is 2 *cm* posterior to the coronal and 2 cm anterior to the lambdoidal suture and 1.5 cm medial to the sagittal suture. This area was identified and marked with a dotted line. Furthermore, this area was divided into five equal proportions (dashed lines) and separated from the skull cap with a band saw. These proportions were used for measurements and reveal the three anatomical layers of a human parietal bone (see Fig. 1b). Measurement locations were marked with an “x”. Thus, 15 measurements were performed on each parietal bone resulting in 30 measurements of each surgical procedure performed on each skull cap.

### Commercially available artificial skull

**Fig. 2:**
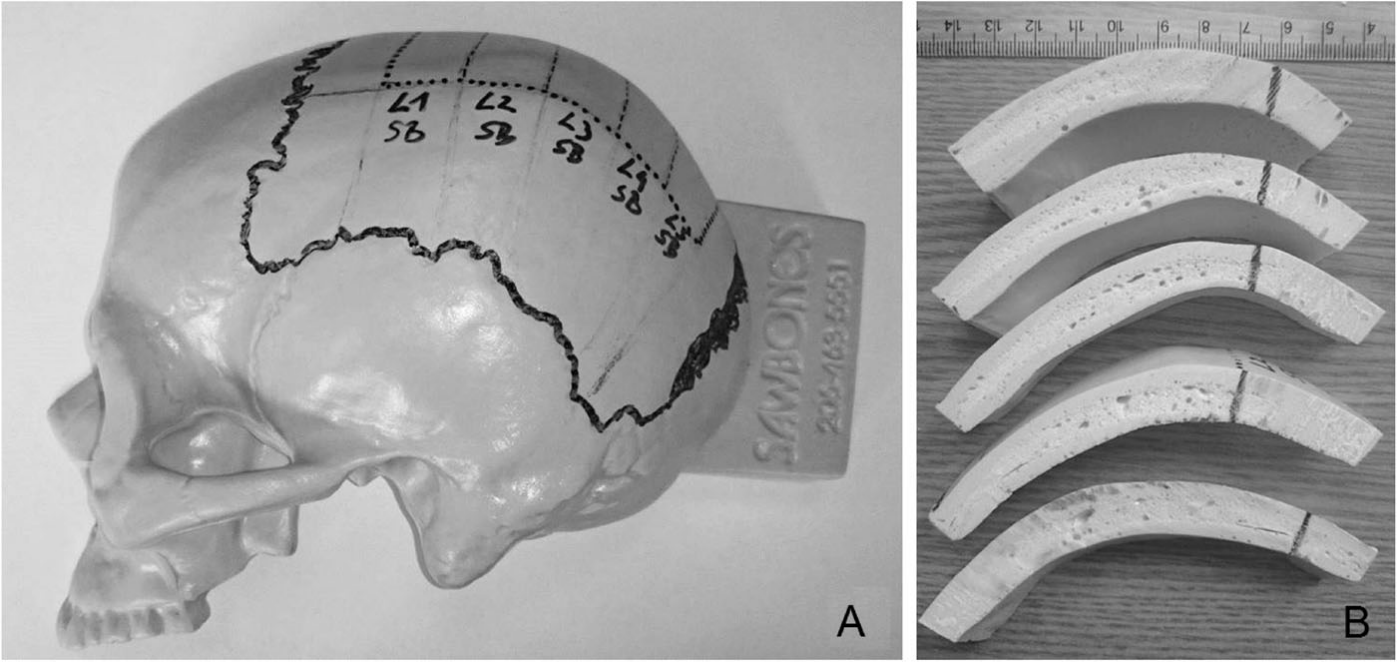
Commercially available skull cap **a** and cut parietal slices of the left hemisphere **b**

Commercially available artificial left partial skulls (*n* = 2, Sawbones, Malmö, Sweden), cut in the sagittal plane and conceited for surgical training were obtained and used as reference. These partial skulls (SB) were made of solid PU foam (density 10PCF, approximately 160 kg/m^3^). Analogue to the aforementioned specimen preparation, the coronal, lambdoidal, squamous and sagittal suture were identified (full thick line, see 2a). Proportions within 1.5 cm from the sagittal suture and 2 cm from the coronal and lambdoidal suture were omitted. The remaining parietal bone was divided into five proportions. The identified bone proportions (dotted line, see Fig. 2a) were marked and cut with a band saw (see Fig. 2b).

### Microcomputed tomography and thickness measurements

**Fig. 3:**
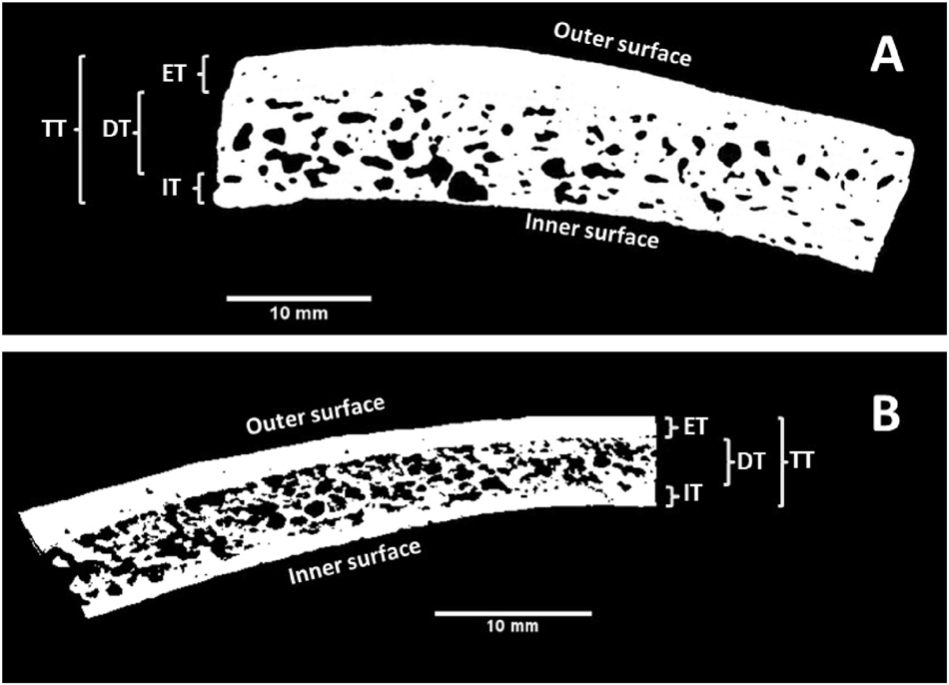
Determination of skull thickness values. **a** Processed µCT image of a human parietal skull sample, **b** Processed photograph of artificial skull cap sample (TT total thickness, ET externa thickness, IT interna thickness, DT diploe thickness)

The human specimens were scanned with a *μ*CT (70 *kVp*, 114*A*, *μ*CT80, Scanco Medical, Brüttisellen, Switzerland) resulting in a slice thickness of 0.09 mm (isometric voxels). The artificial skull pieces were tilted and photographed (resolution 8M, DMC-F/30 with 12x optical mega OIS zoom, Panasonic Corporation, Osaka, Japan) to analyze the bone layer thickness values. The image data obtained from the µCT and the photographs were analyzed using ImageJ (V1.49, National Institutes of Health, Bethesda, USA, [31]). All images were preprocessed according to the settings published by Larsson and colleagues: first, the images were filtered with a three dimensional median filter followed by the setting of a manual color threshold [32]. The total (TT), externa (ET), interna (IT) and diploe (DT) thickness values were measured with the caliper tool in ImageJ (see Fig. 3). For each sample, thickness measurements were performed on three evenly distributed locations. This resulted in *n* = 60 ASC thickness values, *n* = 30 measurement result for the human samples and SB skull, each.

### Machining measurement setup

**Fig. 4:**
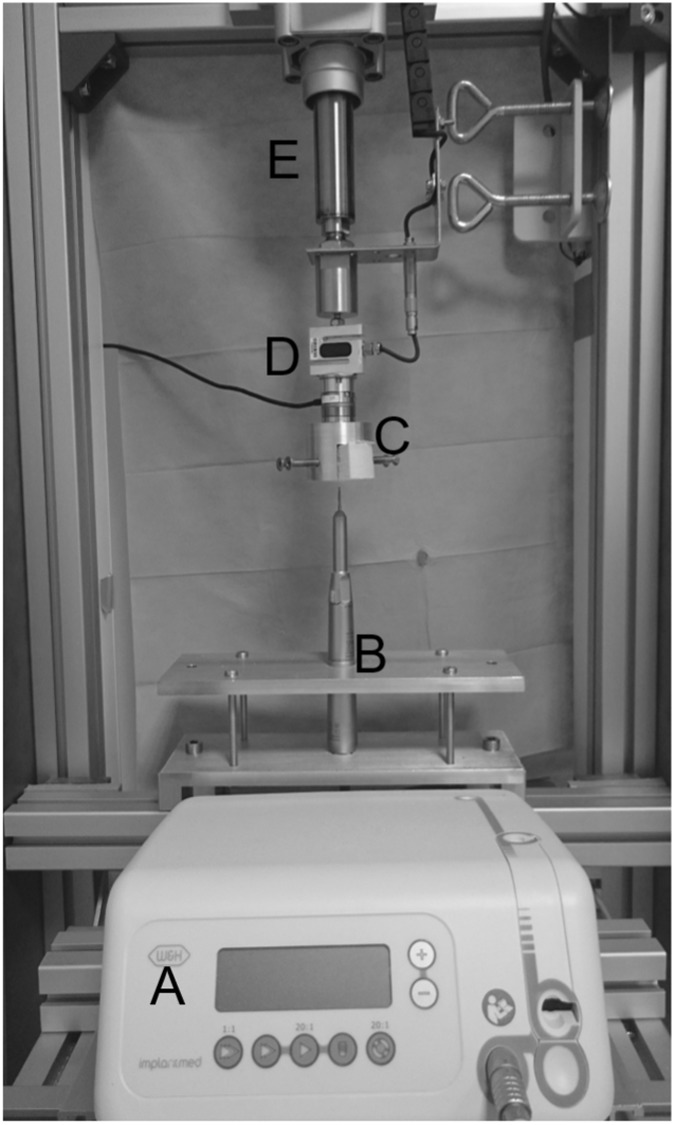
Machining test setup (**a** surgical drive; **b** clamped handpiece; **c** clamped sample; **d** load cell; **e** cylinder rod)

According to the aforementioned graft lift procedure, the maximum insertion forces during drilling and milling of the outer table and during sawing of the diploe were identified as characteristic haptic parameters. The measurement procedure is described in detail elsewhere [28]. A custom made material test bench (see Fig. 4) was designed to move the specimens, which were mounted to a six degree of freedom load cell (axial resolution 1/16*N*, nano25, ATI Industrial Automation, Apex, USA) onto the fast rotating (40,000 rotations per minute) tips of a surgical hand drive (Implantmed SI-923, W&H Dental, Bürmoos, Austria). Axial insertion forces and depth were recorded. The specimens were placed perpendicular in a manner, so that the drill and mill heads were able to penetrate the cortical layer. For the sawing measurements, the specimens were tilted for 90 deg to enable only a penetration of the sawblade into the cancellous diploe. Care was taken, that no cortical structures were included into the sawing measurements. The tools used and the measurement specifications are summarized in Table 1.

**Table 1:** Tools used for surgical machinery measurements and measurement specifications

Procedure	Tool tip	Feed rate (mm/s)	Insertion depth (mm)
Drilling	2 mm drill head (Stryker Corp., Kalamazoo, USA)	1	10
Milling	engraving mill head (Variodent, Neuss, Germany)	1	10
Sawing	10 mm sawblade (W&H Dental, Bürmoos, Austria))	0.5	5

### Human specimens

To determine, if the ASC provided realistic machining haptics, they were compared to results on machining of human sample bones performed in an earlier study([28]): two human parietal bones were released from soft tissue, autoclaved and cut into proportions of 2 × 8 cm. Bone proportions of the temporal, occipital or frontal bone were omitted. Further, the proportion 2 cm lateral of to the sagittal suture was left out during grafting to avoid damage of the underlying sagittal sinus. The human bone pieces were stored at −37 degree Celsius and were defrosted in saline solution at room temperature prior to all measurements. Furthermore, these aforementioned human bone samples were used to examine their structure by µCT measurements within this study.

### Statistical analysis

Statistical analysis was performed using the software SPSS (SPSS Statistics 22, IBM, Armonk, USA). Shapiro- Wilk test for small sample groups was used to test the data for normal distribution. Further, Levene- test was performed to test for homogenous variances. Student’s t-test was used for the testing of differences between groups for normally distributed data with homogenous variances. Non-normally distributed data or data with inhomogeneous variances were tested with Whitney-U test. For all tests, a *p*-value of 0.05 or less was considered significant. Additionally, explorative statistic values (mean values and standard deviations) were calculated.

### Model-based simulator

**Fig. 5:**
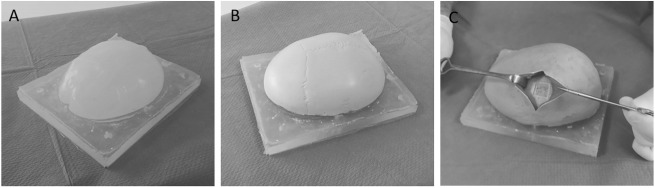
Model-based simulator for parietal graft lift training. **a** skid proof base with a brain-shaped bulge. **b** artificial skull bone cap. **c** internal view of the skull cap edited with artificial soft tissue

A prototype of the model-based simulator was assembled (see Fig. 5). The aforementioned customized artificial skull cap was covered with an artificial skin (dragon skin, Kaupo Plankenhorn e.K., Spaichingen, Germany) and underlying artificial muscles made of red-colored silicone (ecoflex030, Kaupo Plankenhorn e.K., Spaichingen, Germany, [33]). Further, a skid-proof base plate with a brain-shaped bulge made of silicone (ecoflex050, Kaupo Plankenhorn e.K., Spaichingen, Germany) was casted. The brain-shaped bulge served as supporting surface for the artificial skull cap. Further, the artificial brain delivered a change of the machining haptics when the skull cap was completely penetrated with the instruments.

## Results

**Fig. 6:**
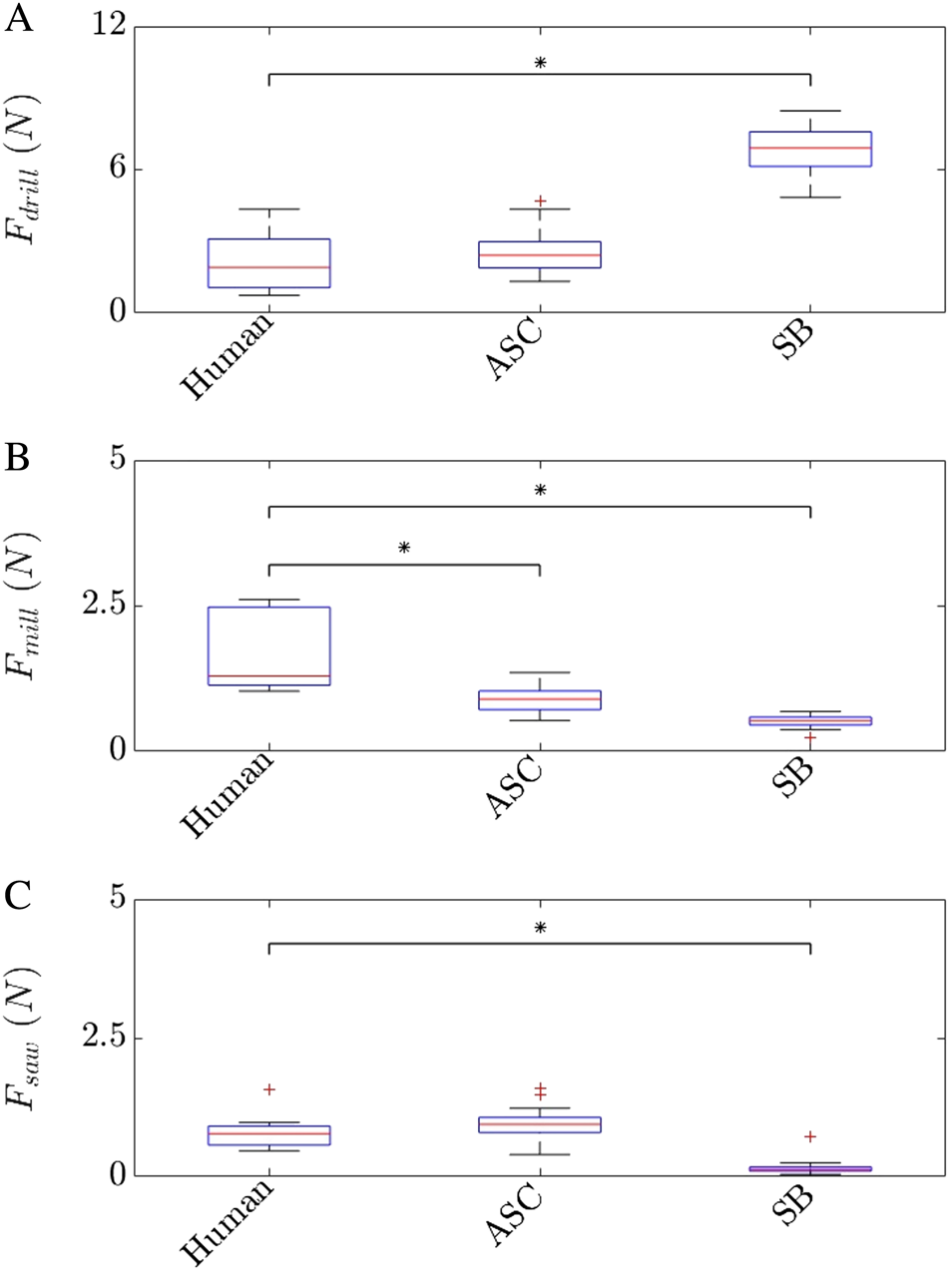
Results of surgical machinery measurements in parietal skulls (Human, [28]) and two artificial skulls (ASC customized artificial skull, SB commercially available partial skull)

Mechanical testing in general revealed non-normal distributions for human milling results [28]. Further, Levene-test detected inhomogeneous variances for ASC drilling and SB sawing results. Thus, Whitney-U-Test was used to assess differences between machining results of human and both artificial bone sample groups (Fig. 6 and Table 2).

**Table 2:** Results of machining measurements in human parietal bone (Human, [28]), customized parietal skull bones (ASC) and a commercially available bone model (SB), (range (minimum to maximum values) and p-value in comparison to human bone)

	Human	ASC		SB	
	min-max	min-max	*p*-value	min-max	*p*-value
Drilling (*N*)	0.74–4.34	1.32–4.69	0.269	4.83–8.47	<0.001
Milling (*N*)	1.40–2.60	0.53–1.35	<0.001	0.23–0.68	<0.001
Sawing (*N*)	0.47–1.57	0.39–1.599	0.178	0.05–0.72	<0.001

### Machining results

#### Drilling

Significant differences were observed between drilling of the SB and the human specimens (*p* < 0.001). The drilling forces for human specimens were 1.8 ± 0.5*N*. The drilling forces for ASC were similar (2.5±, 0.7*N*, *p* = 0.269) while the drilling forces for SB specimens were more than three times larger (6.87 ± 0.96*N*, *p* < 0.001).

#### Milling

Significant differences were observed between milling of the ASC, SB and the human specimens (*p* < 0.001 for both artificial sample groups). The milling forces for the human specimens were 1.7 ± 0.3*N*. The milling forces for SB were about 70% smaller (0.5 ± 0.1*N*) while the milling forces of the ASC were only about 47% smaller (0.9 ± 0.2*N*).

#### Sawing

The sawing forces for the human diploic bone specimens were 0.9 ± 0.1*N*. The sawing forces for ASC were similar (0.9 ± 0.2*N*, *p* = 0.178) while the sawing forces of the SB specimens were more than four times smaller (0.2 ± 0.1*N*, *p* < 0.001).

#### Imaging

**Fig. 7:**
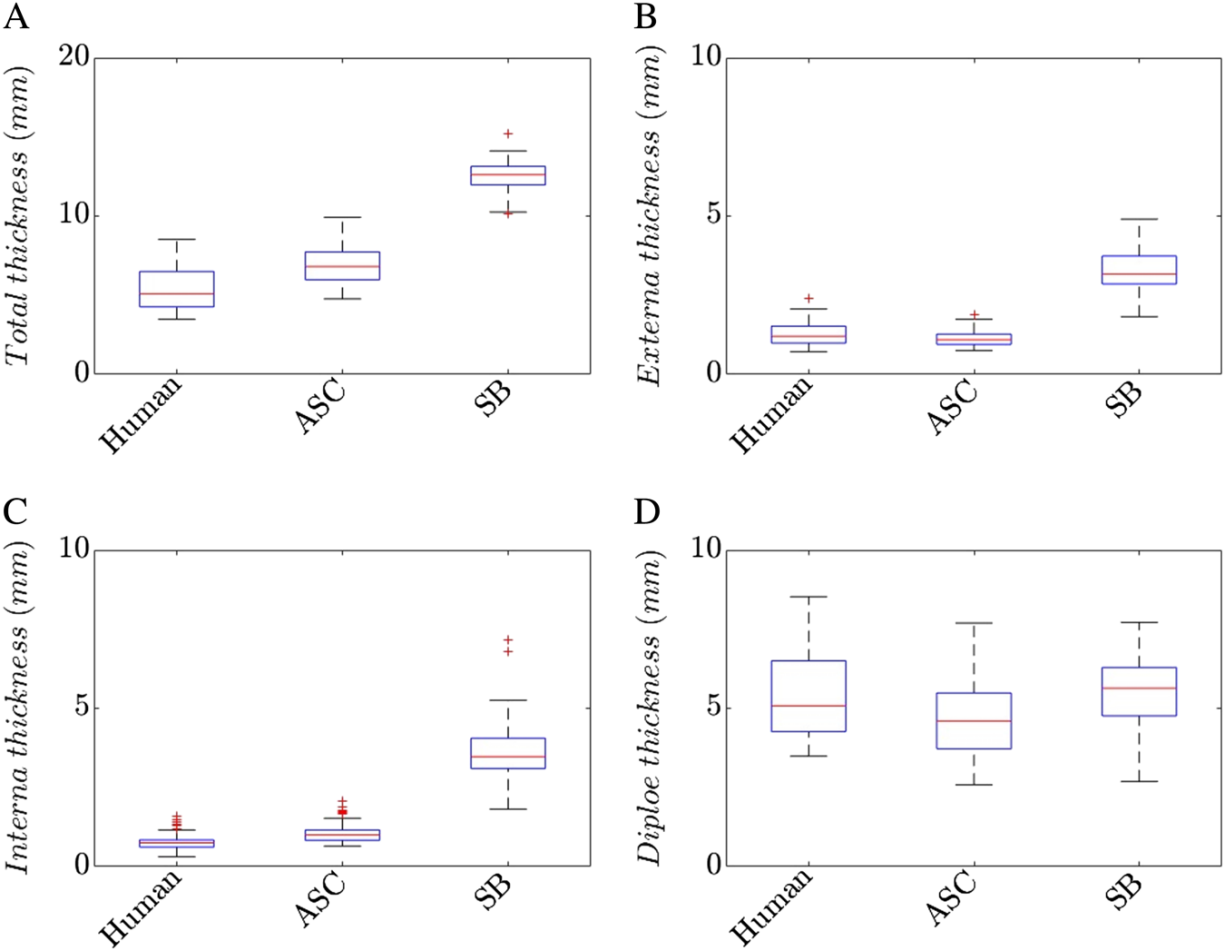
Results of thickness measurements of parietal skulls (Human), a customized skull (ASC) and a commercially available skull (SB)

All imaging results are summarized in Fig. 7. The total thickness values of the human parietal bone samples were 5.31 ± 1.24 mm. The total thickness of the ASC was 6.94 ± 1.20 mm while the SB skull showed a total thickness of 12.51 ± 1.14 mm. The SB samples were more than twice as thick (+135.4%) as the human bone samples while the ASC samples were 30.1% larger. The externa thickness values of the human bones were 1.26 ± 0.34 mm. However, the artificial bones varied up to 9% for the ASC (1.15 ± 0.25 mm) and more than 165% (3.35 ± 0.73 mm) for the SB samples. Similar results were found for the interna thickness values. The average interna thickness of the human parietal bone was 0.76 ± 0.22 mm. ASC (1.07 ± 0.34 mm, +40.7%) and SB (3.69 ± 1.18 mm, +385.5%) were significantly thicker. The measured thickness values of the diploic space were 4.72 ± 1.09 mm for the ASC samples, 5.45 ± 1.18 mm for the SB samples and only 3.28 ± 0.95 mm for the human samples. Thus, the diploe of the artificial skull bone samples were 43.9% (ASC) and 66.2% (SB) thicker than the human bone. Statistical tests revealed significant differences between human and artificial groups for all thickness values.

### Model-based simulator

**Fig. 8:**
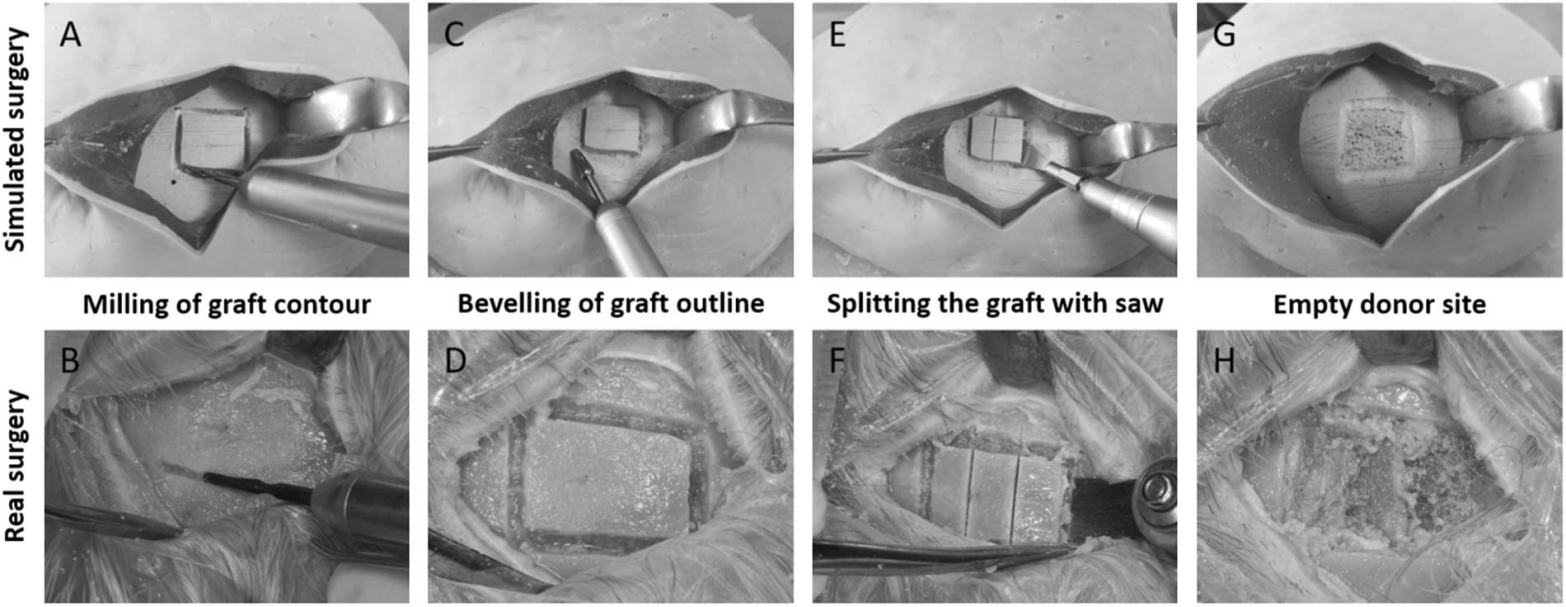
Procedural steps of a tabula externa graft lift surgery. Upper image sequence: simulated surgery; lower image sequence: real surgery.; **a**, **b** milling of a “bone island”, **c**, **d** flattening of graft-outlines, **e**, **f** cutting diploic connections, **g**, **h** empty donor site

A prototype of the model-based simulator was independently tested by two experienced oral and maxillofacial surgeons. The image sequences in Fig. 8 oppose various steps of a parietal graft lift surgery performed at human skull (lower image sequence) and the novel model-based simulator (upper image sequence). The main steps, namely contour-milling of the graft, bevelling of the grafts' outline, splitting of the graft with a saw and the final lifting of the graft with a chisel could be realistically performed with the model-based simulator, as reported by the surgeons. Further, the initial incision into the artificial scalp to get access to the underlying bone was congruently described as realistic. Additionally, the opened scalp needed to be retracted in order to augment the limited access to the skull bone, a manouvre comparable and necessary in almost all authentic procedures.

## Discussion

A well-designed parietal bone model, which was validated against human parietal bones, was presented in this study. Such a model-based simulator will provide a realistic training platform useful for education and training of surgical novices. Training at this model can potentially reduce intrasurgical complications due to a lack of appropriate handling skills with the fast rotating and oscillating surgical drives.

The purpose of this study was to validate the surgical machining properties of an already available PU mixture which mimics the properties of a human parietal bone. In contrast to an earlier study [28], the material mixture was molded with an additional technique to receive different bone-mimicking layers imitating a human parietal bone. Although three dimensional printing models promise a variety of medical applications, just like surgical planning, teaching and practicing, this quite cheap and easy manufacturing technology was not considered for the manufacturing of our ASC, yet. Some trials were performed to 3D print the cortical layer and further fill the cortical shell with our polyurethane foam mixture, but the mechanical testing results were not satisfying. The 3D printed cortical layers did not bind properly to the poylurethane foam. During milling and the chiseling of the created graft, the cortical layer splintered off, but during this surgical procedure it is necessary not to lift the cortical layer alone but also harvest a part of the underlying cancellous diploe. A few limitations of this study have to be mentioned. The PU- mixtures used are not consistent with the ASTM F1839 standard. This standard designates different density grades, where the largest grade 50 corresponds to a material density of 800 kg/m^3^ [22]. This density was not met with the used PU base material, which revealed a material density of greater than 1000 kg/m^3^. Further morphometric parameters of the PU foam imitating the cancellous layer of the skull are not determined so far. A detailed analysis is part of ongoing research. Although the human sample size used here was rather small (*n* = 2 parietal bones) and samples stem from female donors over the age of 65, earlier studies clearly demonstrated that the total parietal thickness does not increase with age [5]. Since further, statistical differences in total thickness between men and women were not observed [5] and since parietal bone thickness and morphometrics are not affected by age and osteoporosis [34, 35] the small sample size was considered as reliable and meaningful. Human bone samples were autoclaved prior to the measurements which potentially could alter their biomechanical properties. Indeed, changes of the bone`s biomechanical properties following autoclaving have been observed and revealed a reduction of the Young's modulus of about 47% and a 48% reduction in bone strength while the density apparently remained unaltered [36]. An investigation by Voggenreiter et al., who examined the changes of the surface structure of cortical bones due to autoclaving, found no adverse effects in density or structure [37]. However, changes of the application forces due to surgical machining of autoclaved bone are not available yet and need to be investigated in upcoming studies. The measured thickness values of the human parietal bone samples were in accordance to the literature. The total thickness of parietal skulls was investigated by Sabancıoğulları et al., reporting a total thickness of 6.69 ± 1.94 mm [38]. Parietal externa thickness was investigated by Peterson et al. and Jung et al., reporting thickness values of 1.6 ± 0.2 mm [7] and 1.8 ± 0.3 mm [39]. Peterson et al. also examined the thickness of the inner parietal cortices and reported an average thickness of 1.7 ± 0.3 mm [39]. A diploic thickness of 3.38 ± 1.00 mm of human parietal bones was reported by Hatipoglu et al. [40]. In summary, the results are in line with earlier data.

The measured thickness values of the ASC were all within the range of the obtained human data or the aforementioned literature data. Thus, the created artificial skull cap was suitable to mimic human bone layers realistically. The measured total thickness of the SB skull was more than two times thicker than the measured total thickness of the human references. The externa thickness and the diploe thickness also were clearly out of the range of the human bone samples and most likely contribute to the different mechanical properties observed (see below). The interna thickness values of SB were more than four times higher than the human ones. However, a special attention should be drawn to the thickness of the externa layer, which is lifted during the split graft lift. The forces measured during the drilling of the SB specimen were twice as high as the ones obtained from human reference bones. The statistical analysis detected significant differences between the human bones and the SB samples. These high forces may result from the outsized thickness of the outer table. The maximum insertion force arose from an axial puncture force of the drilling tip in combination with frictional forces of the flutes and helices of the drill bit when passing the cortical layer. Thus, the maximum puncture force arose when the drill tip broke through the cortical layer and was followed by a sharp decrease of the drilling force during the entering of the diploic space. The drilling forces of ASC were similar to the human ones. During the contouring of the graft with a mill head, experienced surgeons normally hold the surgical handpiece in an oblique position (e.g.like a pen is used). This inclined position of the tool allows the surgeon to “pull” the mill through the cortical layer to form a trough. Novice surgeons are instructed to hold the drive upright and to insert the milling head perpendicularly into the cortical layer of the calvarial bone. To contour the graft, one hole after another is milled. As the final milling step, the drive shall be hold more oblique and the remaining cortical connections are intersected by pulling the millhead from hole to hole. Because of this teaching experience, the milling forces of the human and artificial skulls were also recorded in an perpendicular approach. Nevertheless, the milling forces of the ASC were significantly different to the human ones. Thus, to improve cortical layer for milling, further investigations are necessary. The sawing results showed, that the maximum sawing forces occurred when all saw teeth were inserted into the cancellous bone. This happened at an insertion depth of approximately 2 mm due to their semicircular arrangement. Due to the addition of the mineral filler the hardness of the open cell PU foam could be increased and thus the custom made diploe was able to deliver comparable haptic sawing feedback like human diploic bone. The prototype of the novel model based simulator was successfully able to mimic all procedural machining steps of a “split thickness graft lift” procedure including the incision and retraction of the artificial scalp. Evidence of face, content and construct validity of the novel simulator is part of ongoing research.

## Conclusion

Concluding, a new parietal skull model suitable for parietal graft lift training was fabricated and validated against human parietal bones. The three step molding process enabled the manufacturing of an artificial skull cap with anatomic realistic layers of the parietal bone proportion. The drilling of the outer table and the sawing of the diploic space resulted in a realistic tactile feedback in comparison to the human reference. However, further investigations are necessary to improve the haptics during milling. The surgical training on the SB model is not recommended. The unrealistic dimension of the externa layer at the SB skull, which was in the range of the total human skull thickness, is unsuitable for a novice surgeon to train a parietal graft lift. The oversized cortical layer would be teaching a wrong anatomy of the parietal skull and hence would lead to a wrong acquisition of surgical skills.
